# Pediatric Severe Sepsis Checklist Improves Timeliness of Treatment: A Quality Improvement Project

**DOI:** 10.1097/pq9.0000000000000526

**Published:** 2021-12-16

**Authors:** Sarah J. Maciolek, Emily C. Dawson

**Affiliations:** From the Pediatric Emergency Department, Advocate Children’s Hospital, Oak Lawn, Ill.

## Abstract

The Children’s Hospital Association’s Improving Pediatric Sepsis Outcomes (IPSO) collaborative is a multi-center quality improvement (QI) learning collaborative of 61 U.S. children’s hospitals that seeks to improve sepsis outcomes through collaborative learning and reliable implementation of evidence-based interventions in pediatric emergency departments, intensive care units, general care units, and hematology/oncology units. Specifically, IPSO’s goals are to decrease sepsis-attributable mortality and prevent hospital-onset sepsis among children.

The following 10 abstracts represent a select group of projects undertaken by IPSO participating hospitals that were presented at one of three collaborative events in 2020 and 2021. IPSO’s Research Workgroup reviewed all submitted abstracts and selected the top 10 for inclusion in this Supplement

## Introduction:

Priorities of care within the first-hour resuscitation bundle for pediatric severe sepsis include rapid establishment of vascular access, fluid resuscitation, and initiation of empiric antimicrobial therapy. The utilization of a protocolized treatment in the Emergency Department (ED) is associated with improved timeliness of care and reduced morbidity related to organ dysfunction. Despite implementation of an order set, the Pediatric EDs within a multi-site hospital system failed to consistently meet established goals of the severe sepsis resuscitation bundle. The purpose of this Quality Improvement project is to decrease time to antibiotic and fluid resuscitation for children presenting to the ED with severe sepsis through implementation of a checklist.

## Methods:

The team utilized the Plan-Do-Study-Act model for process improvement to implement a pediatric severe sepsis checklist. An interprofessional team developed the nurse-driven checklist outlining the essential elements in the first hour of care. All ED care providers completed computer-based training highlighting utilization of the checklist in the clinical setting. Process improvement measures were collected using completed checklists and electronic medical records.

## Results:

**Fig. 1.**
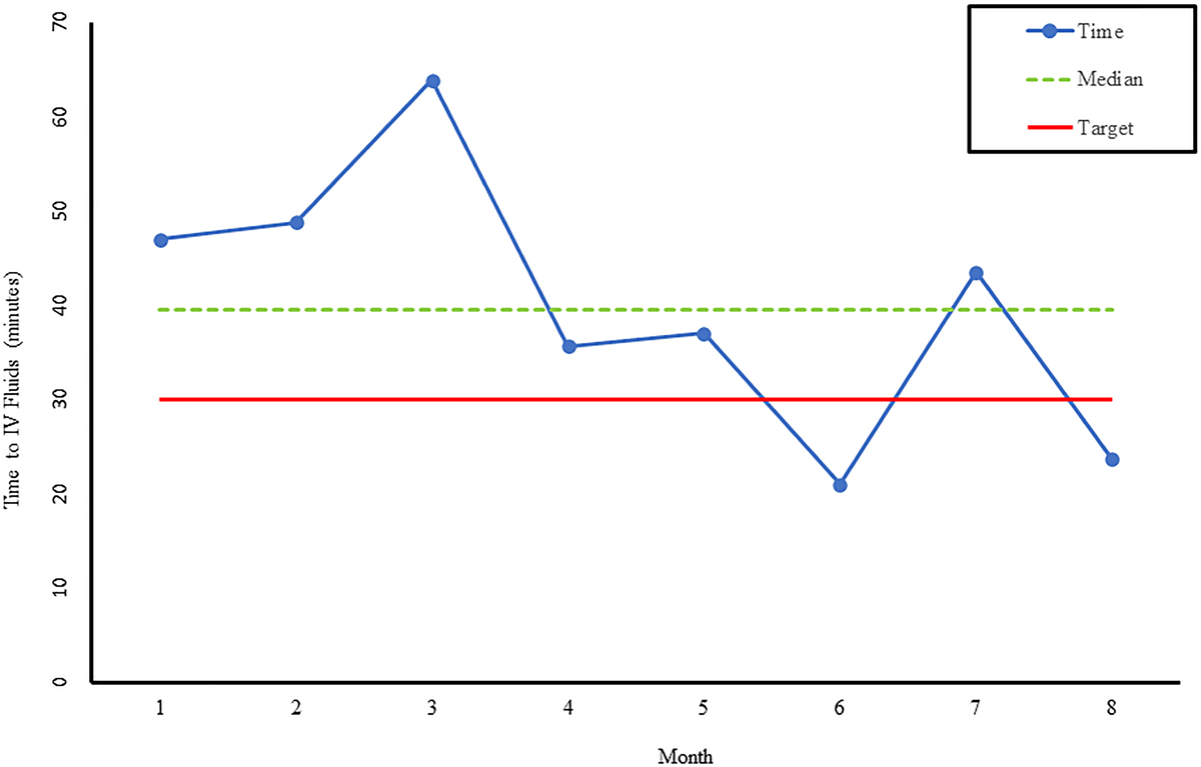
Graph of average time to IV fluid administration from recognition for children presenting with severe sepsis. Months 1–3 represent performance prior to implementation of pediatric severe sepsis checklist. Months 4–8 represent performance after checklist implementation.

**Fig. 2.**
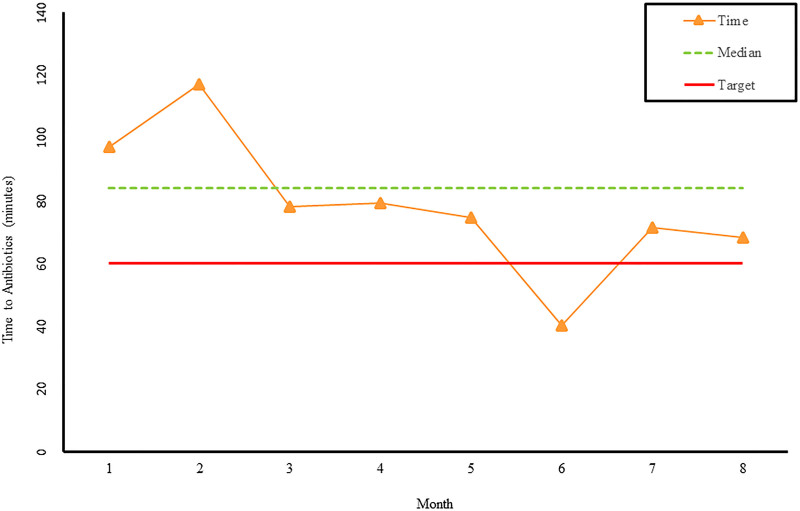
Graph of average time to antibiotic administration from recognition for children presenting with severe sepsis. Months 1–3 represent performance prior to implementation of pediatric severe sepsis checklist. Months 4–8 represent performance after checklist implementation.

The checklist was utilized in 54% of the severe sepsis events postimplementation. Time to fluid resuscitation and antibiotics improved. Average time to fluid bolus delivery decreased from 52 to 30 minutes (Fig. 1). Average time to antibiotic delivery decreased from 101 to 60 minutes (Fig. 2). Further improvements were noted when the checklist was utilized. Order set utilization increased from 25% to 58%.

## Conclusion:

Despite less optimal use of the checklist during severe sepsis events, implementation of a checklist led to improved process outcomes.

